# STING induces LC3B lipidation onto single-membrane vesicles via the V-ATPase and ATG16L1-WD40 domain

**DOI:** 10.1083/jcb.202009128

**Published:** 2020-11-17

**Authors:** Tara D. Fischer, Chunxin Wang, Benjamin S. Padman, Michael Lazarou, Richard J. Youle

**Affiliations:** 1 Biochemistry Section, Surgical Neurology Branch, National Institute of Neurological Disorders and Stroke, National Institutes of Health, Bethesda, MD; 2 Department of Biochemistry and Molecular Biology, Biomedicine Discovery Institute, Monash University, Melbourne, Australia

## Abstract

Following the detection of cytosolic double-stranded DNA from viral or bacterial infection in mammalian cells, cyclic dinucleotide activation of STING induces interferon β expression to initiate innate immune defenses. STING activation also induces LC3B lipidation, a classical but equivocal marker of autophagy, that promotes a cell-autonomous antiviral response that arose before evolution of the interferon pathway. We report that STING activation induces LC3B lipidation onto single-membrane perinuclear vesicles mediated by ATG16L1 via its WD40 domain, bypassing the requirement of canonical upstream autophagy machinery. This process is blocked by bafilomycin A1 that binds and inhibits the vacuolar ATPase (V-ATPase) and by SopF, a bacterial effector that catalytically modifies the V-ATPase to inhibit LC3B lipidation via ATG16L1. These results indicate that activation of the cGAS-STING pathway induces V-ATPase–dependent LC3B lipidation that may mediate cell-autonomous host defense, an unanticipated mechanism that is distinct from LC3B lipidation onto double-membrane autophagosomes.

## Teaser

A primordial function of the cGAS/STING pathway may be to induce LC3B lipidation. Fischer et al. show cGAMP-activated STING induces the V-ATPase to recruit ATG16L1 for lipidation of LC3B onto single-membrane perinuclear vesicles. The bacterial effector SopF blocks this pathway, suggesting roles in host defense.

## Introduction

Host pathogen defense involves innate immune responses encompassing cell-nonautonomous and cell-autonomous pathways to alert professional immune cells and eliminate pathogens from within infected cells, respectively. Autophagy is an evolutionarily conserved intracellular degradation process involving the de novo formation of a double-membrane vesicle, the autophagosome, that sequesters cellular content and delivers it to the lysosome for degradation (Morishita and Mizushima, 2019). Autophagosomes can form to recycle bulk cytoplasmic material for nutrient replenishment or more selectively to degrade damaged organelles or intracellular pathogens, such as bacteria (known as xenophagy), providing a cytosolic defense mechanism that can facilitate cell-autonomous innate immunity (Randow et al., 2013). Autophagy involves several multi-subunit protein complexes to drive the nucleation of a cup-shaped phagophore, its elongation, and closure into a double-membrane vesicle, and the final fusion of the autophagosome with the lysosome (Melia et al., 2020). In mammals, the nucleation of the phagophore is initiated by the Unc51-like kinase (ULK) complex (ULK1/2, FIP200, ATG13, and ATG101) and ATG9-containing vesicles. ULK1 kinase activity recruits the phosphoinositide 3-kinase (PI3K) complex (Vps34, Beclin-1, ATG14, and Vps15) to generate phosphatidylinositol 3-phosphate (PI3P) on growing phagophores, which recruits WD repeat domain phosphoinositide-interacting proteins (WIPIs) and DFCP1 via their PI3P binding domains. WIPI2 then binds ATG16L1 to promote conjugation of mammalian ATG8 proteins (LC3A, B, and C, and GABARAPL1 and L2) to phosphatidylethanolamine by the ATG12-ATG5-ATG16L1 complex in an E3 ubiquitin ligase–like manner on both the convex and concave membrane surfaces of phagophores. Lipidation of ATG8s, specifically LC3B, is a hallmark of autophagosome formation and a common marker to detect autophagy but has also been observed to occur on membranes independently of autophagy (Heckmann and Green, 2019).

Intracellular bacteria are detected by galectins and autophagy receptors (NDP52, optineurin, and p62) that initiate autophagosome formation at vacuoles containing bacteria that become damaged during infection (Thurston et al., 2009, 2012; Wild et al., 2011; Zheng et al., 2009). Additionally, pathogens can be detected by pattern recognition receptors (PRRs) that sense pathogen-associated molecular patterns, such as nucleic acids or cell wall components, to initiate transcription of innate immune genes (Lopes Fischer et al., 2020). Stimulator of interferon genes (STING) is a conserved component of an innate immune pathway that initiates type I interferon (IFN) signaling following the detection of foreign DNA in the cytosol by cyclic guanosine monophosphate (GMP)-adenosine monophosphate (AMP) synthase (cGAS; Ahn and Barber, 2019; Chen et al., 2016). Upon detection of DNA in mammals, cGAS synthesizes the cyclic dinucleotide 2′3′-cGMP-AMP (cGAMP), which binds directly to STING to initiate its trafficking from the ER to the Golgi apparatus and downstream transcription of IFNs. Recently, activation of STING by cGAMP has been found to also induce the lipidation of LC3B (Gui et al., 2019). cGAMP-induced LC3B lipidation was associated with limiting intracellular viral replication independently of the IFN response, indicating that autophagy induction may be an additional innate immune function of the cGAS-STING pathway (Gui et al., 2019; Yamashiro et al., 2020). Interestingly, LC3B lipidation induced by cGAMP was also found to be a conserved function of a STING homologue in an organism that predates the appearance of the IFN response, *Nematostella vectensis*, presenting autophagy as a potential primordial function of STING to facilitate cell-autonomous innate immunity (Gui et al., 2019; Kranzusch et al., 2015; Margolis et al., 2017).

Although the cGAS-STING pathway has been previously associated with various autophagy machinery (Konno et al., 2013; Liang et al., 2014; Prabakaran et al., 2018; Saitoh et al., 2009; Watson et al., 2012, 2015), Gui et al. (2019) found that LC3B lipidation induced by the activation of STING does not require the key components, ULK1/2 of the ULK complex, VPS34 and Beclin1 of the PI3K complex, or ATG9. As these proteins are known to be required for canonical LC3B lipidation and autophagosome formation, the molecular mechanisms mediating autophagy induced by STING activation remain unclear. Here, we confirm that LC3B lipidation induced by cGAMP activation of STING is independent of most canonical autophagy machinery. We find that LC3B is associated with single-membrane perinuclear vesicles, more so than double-membrane autophagosomes, and its lipidation is mediated by the WD40 domain of ATG16L1. Additionally, cGAMP induced LC3B lipidation and perinuclear foci formation is sensitive to inhibition of the vacuolar (V)-ATPase by bafilomycin A1 (BafA1). BafA1 also blocks ATG16L1 translocation to and redistribution of the V1 complex of the V-ATPase in the perinuclear region induced by cGAMP. Furthermore, the bacterial effector SopF, which targets the V-ATPase to block its interaction with the WD40 domain of ATG16L1 and LC3B lipidation (Xu et al., 2019), also blocks STING-dependent LC3B lipidation and perinuclear foci formation. These data further distinguish STING-dependent LC3B lipidation from the canonical autophagy pathway and suggest a potentially novel mechanism of cell-autonomous host defense mediated by STING.

## Results and discussion

**Figure 1.**
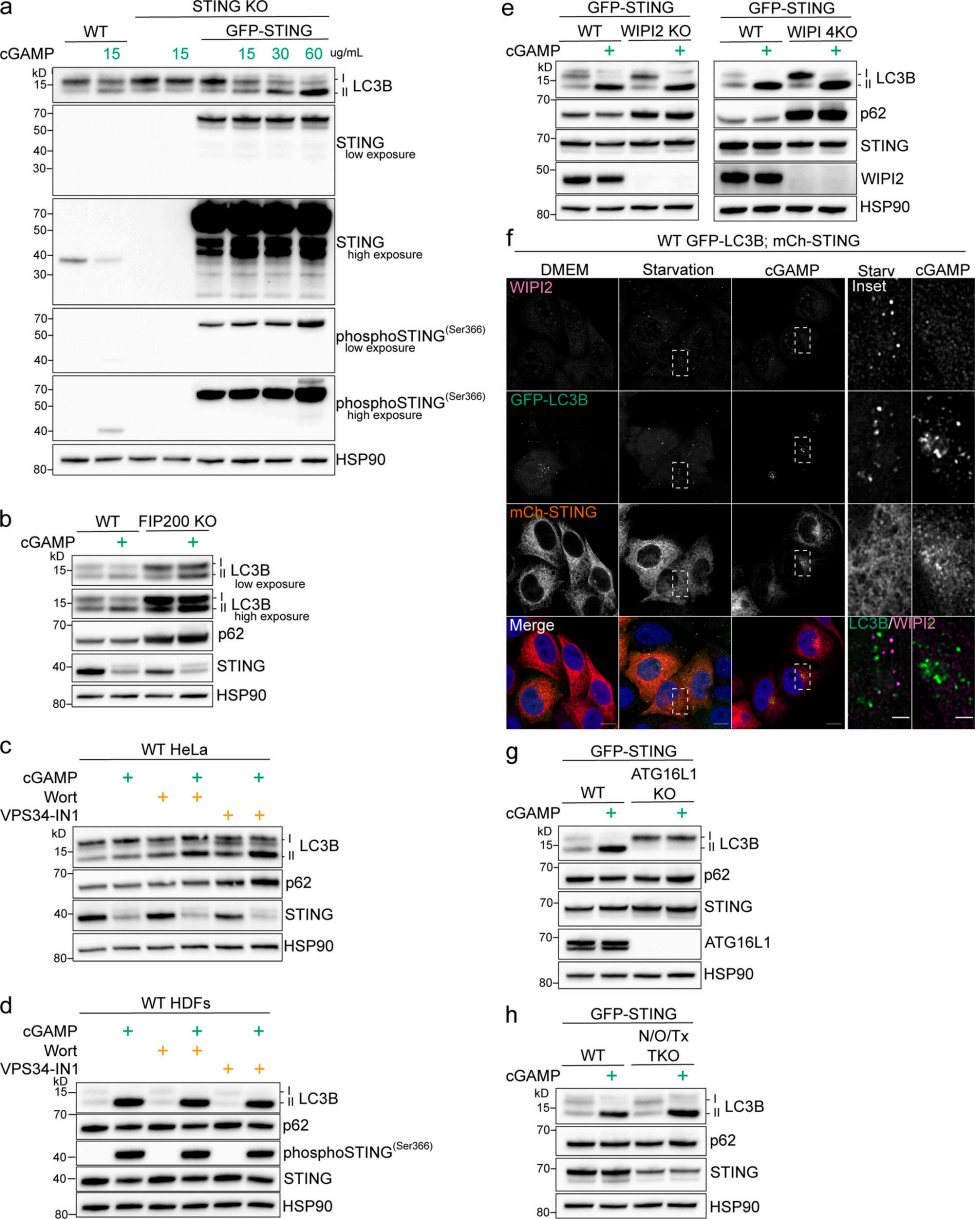
**Characterization of autophagy machinery required for STING-dependent LC3B lipidation. (a)** WT, STING KO, and STING KO HeLa cells stably expressing GFP-STING were treated with 15 µg/ml or the indicated concentrations of cGAMP for 8 h in normal DMEM. **(b)** WT and FIP200 KO HeLa cells were treated with 15 µg/ml cGAMP for 8 h in normal DMEM (all the following experiments were treated the same unless specified). **(c)** WT HeLa cells were treated with cGAMP alone or with cGAMP and wortmannin (200 nM) or VPS34-IN1 (300 nM) for 8 h. **(d)** Primary HDFs were treated with 60 µg/ml cGAMP and wortmannin or VPS34-IN1 at the same concentrations as in c for 4 h. **(e)** WT, WIPI2 KO, and WIPI 4KO HeLa cells stably expressing GFP-STING were treated with 60 µg/ml cGAMP. **(f)** Representative Airyscan-processed confocal imaging of WT HeLa cells stably expressing GFP-LC3B (green) and mCh-STING (red) were treated with 60 µg/ml cGAMP or starvation media (HBSS with Ca^2+^ and Mg^2+^) for 8 h before immunofluorescent labeling of endogenous WIPI2 (magenta). Scale bar, 10 µm, 2 µm (inset). **(g and h)** WT, ATG16L1 KO, and NDP52 (N), optineurin (O), and TAX1BP1 (Tx) triple KO (N/O/Tx TKO) HeLa cells stably expressing GFP-STING were treated with 60 µg/ml cGAMP. Each Western blotting experiment was independently replicated three times. Starv, starvation; Wort, wortmannin.

**Figure S1.**
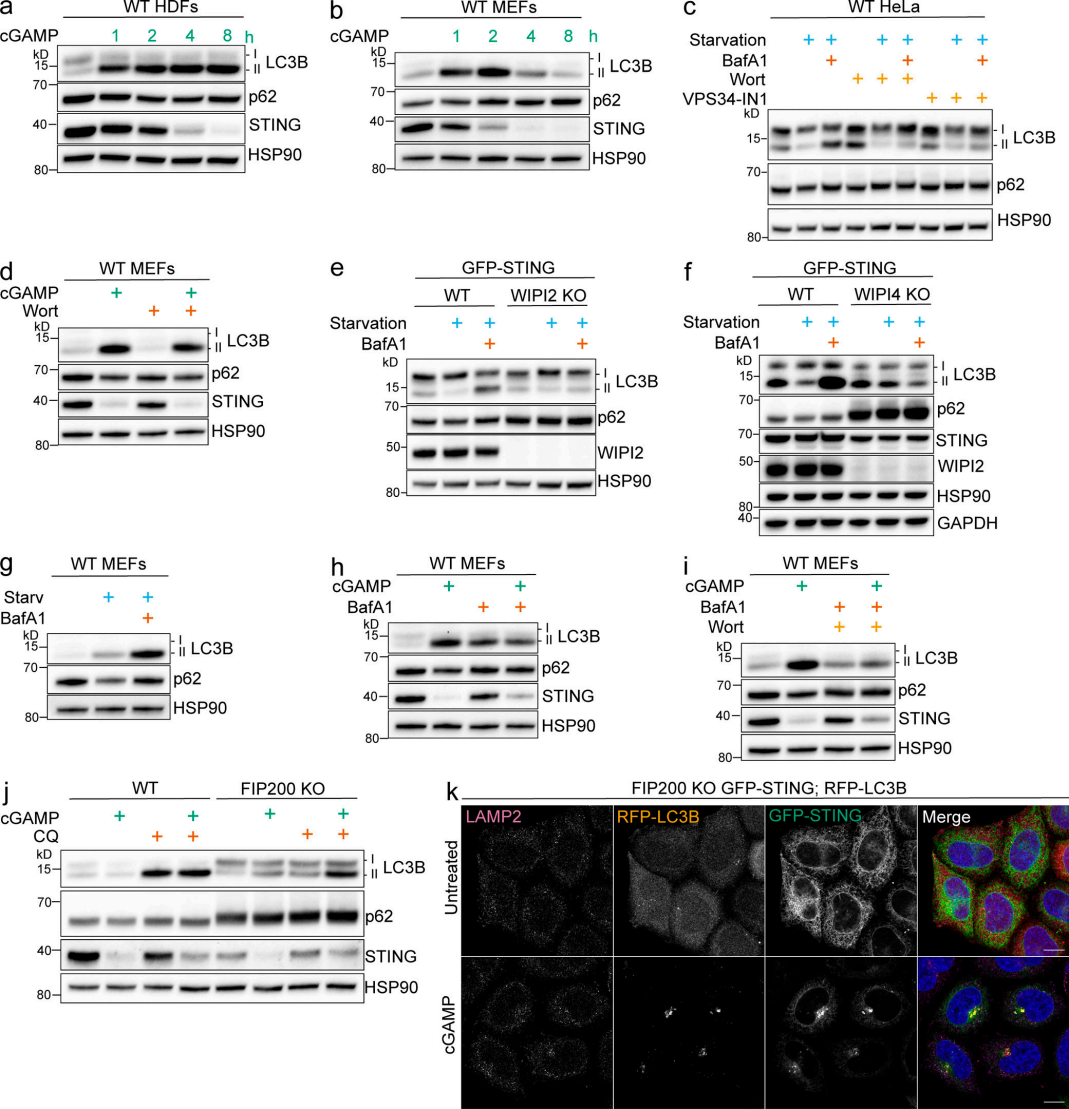
**Experiments supplementary to Figs. 1 and 2.** **(a and b)** Primary HDFs (a) and primary MEFs (b) were treated with 60 μg/ml cGAMP, and cell lysates were collected at the indicated time points. **(c)** WT HeLa cells were incubated in starvation media (HBSS with Ca^2+^ and Mg^2+^) alone and with 100 nM BafA1 and with or without wortmannin (200 nM) or VPS34-IN1 (300 nM) for 4 h. All the following experiments were treated the same unless specified. **(d)** MEFs were treated with 60 μg/ml cGAMP alone and with wortmannin for 2 h. All the following experiments using MEFs use the same conditions. **(e and f)** WT, WIPI2 KO (e), and WIPI 4KO (f) HeLa cells stably expressing GFP-STING were incubated in starvation media alone and with BafA1 for 4 h. **(g)** MEFs were incubated in starvation media alone and with BafA1 for 4 h.** (h and i) **MEFs were incubated with cGAMP alone and with BafA1 (h) or with BafA1 and wortmannin (i). **(j)** WT and FIP200 KO HeLa cells were incubated with 15 µg/ml cGAMP alone and with 20 μM chloroquine (CQ) for 8 h. **(k)** Representative Airyscan-processed confocal imaging of FIP200 KO HeLa cells with stable expression of RFP-LC3B and GFP-STING treated with cGAMP (60 µg/ml) for 8 h, fixed, and immunostained for endogenous LAMP2. Scale bar, 10 µm. Western blotting experiments were independently replicated two (b, c, e, and g) or three (a, d, f, h, i, and j) times. Starv, starvation; Wort, wortmannin.

To explore how STING activation induces LC3B lipidation independently of the ULK and PI3K complexes, we examined the requirement of other key autophagy components involved in LC3B lipidation induced by 2′3′-cGAMP activation of STING. As reported by Gui et al. (2019), we confirmed in HeLa cells that cGAMP treatment induces LC3B lipidation and that this response is completely blocked in STING knockout (KO) HeLa cells (Fig. 1 a). Stable expression of GFP-STING in STING KO cells rescues cGAMP-induced LC3B lipidation, which increases in a cGAMP dose-dependent manner (Fig. 1 a). Degradation of endogenous STING and TBK1 phosphorylation of STING at serine 366 upon cGAMP treatment can also be observed in WT HeLa cells as previously reported, indicating that STING activation and signaling are intact in HeLa cells (Fig. 1 a; Gonugunta et al., 2017; Gui et al., 2019; Liu et al., 2015; Tanaka and Chen, 2012). Degradation of constitutively overexpressed GFP-STING is not observed (Fig. 1 a). cGAMP-induced LC3B lipidation and degradation of endogenous STING can also be observed in primary human dermal fibroblasts (HDFs) and primary mouse embryonic fibroblasts (MEFs; Fig. S1, a and b). Consistent with the finding that LC3B lipidation mediated by STING activation is independent of ULK1/2 (Gui et al., 2019), KO of FIP200, a required component for the initiation step of autophagosome formation by the ULK complex (Hara et al., 2008), did not prevent cGAMP-induced LC3B lipidation (Fig. 1 b). Basal accumulation of p62 is more evident in FIP200 KO compared with WT cells, consistent with a block in basal autophagy (Fig. 1 b). Endogenous STING degradation after cGAMP treatment is not blocked in FIP200 KO cells (Fig. 1 b). The broad specificity PI3K inhibitor, wortmannin, and the selective class III PI3K inhibitor, VPS34-IN1, prevented serum and amino acid starvation–induced LC3B lipidation (Fig. S1 c), but did not prevent cGAMP-induced LC3B lipidation in WT HeLa cells, primary HDFs, or primary MEFs (Fig. 1, c and d; and Fig. S1 d), consistent with the previously reported nonessential function of Vps34 upstream of cGAMP-induced LC3B lipidation (Gui et al., 2019). Despite LC3B lipidation without PI3P generation by PI3Ks, the PI3P effector, WIPI2, has been reported to be a required component for cGAMP-induced LC3B lipidation in BJ cells (immortalized human fibroblasts; Gui et al., 2019). However, WIPI2 KO in HeLa cells did not prevent LC3B lipidation with cGAMP treatment (Fig. 1 e, left panel). To address the potential compensation by other WIPIs in HeLa cells, we knocked out all four human isoforms of WIPI (WIPI1-4; WIPI 4KO). KO of all four WIPIs robustly blocked starvation-induced LC3B lipidation, similar to the KO of WIPI2 (Fig. S1, e and f), but did not block cGAMP-induced LC3B lipidation (Fig. 1 e, right panel). As in FIP200 KO cells (Fig. 1 b), basal accumulation of p62 is observed in both WIPI2 KO and WIPI 4KO compared with WT cells, consistent with a block in basal autophagy (Fig. 1 e). Additionally, we assessed whether endogenous WIPI2 forms punctae induced by cGAMP in WT HeLa cells stably expressing GFP-LC3B and mCherry (mCh)–STING (Fig. 1 f). Increased WIPI2 punctae formation can be observed upon amino acid and serum starvation; however, WIPI2 punctae are not apparent above basal levels and do not colocalize with perinuclear LC3B foci upon exposure to cGAMP (Fig. 1 f). Collectively, these data indicate that LC3B lipidation induced by STING activation is independent of WIPI2 in HeLa cells. Further, consistent with the requirement of ATG5 (Gui et al., 2019), KO of ATG16L1, a key component of the ATG5-12-16L1 complex required for ATG8 conjugation, blocked cGAMP-induced LC3B lipidation (Fig. 1 g). Combined triple KO (TKO) of the autophagy receptors, NDP52 (N), optineurin (O), and TAX1BP1 (Tx; N/O/Tx TKO) in HeLa cells did not prevent cGAMP-induced LC3B lipidation (Fig. 1 h), indicating that STING activation does not induce LC3B lipidation similarly to xenophagy and mitophagy x pathways.

**Figure 2.**
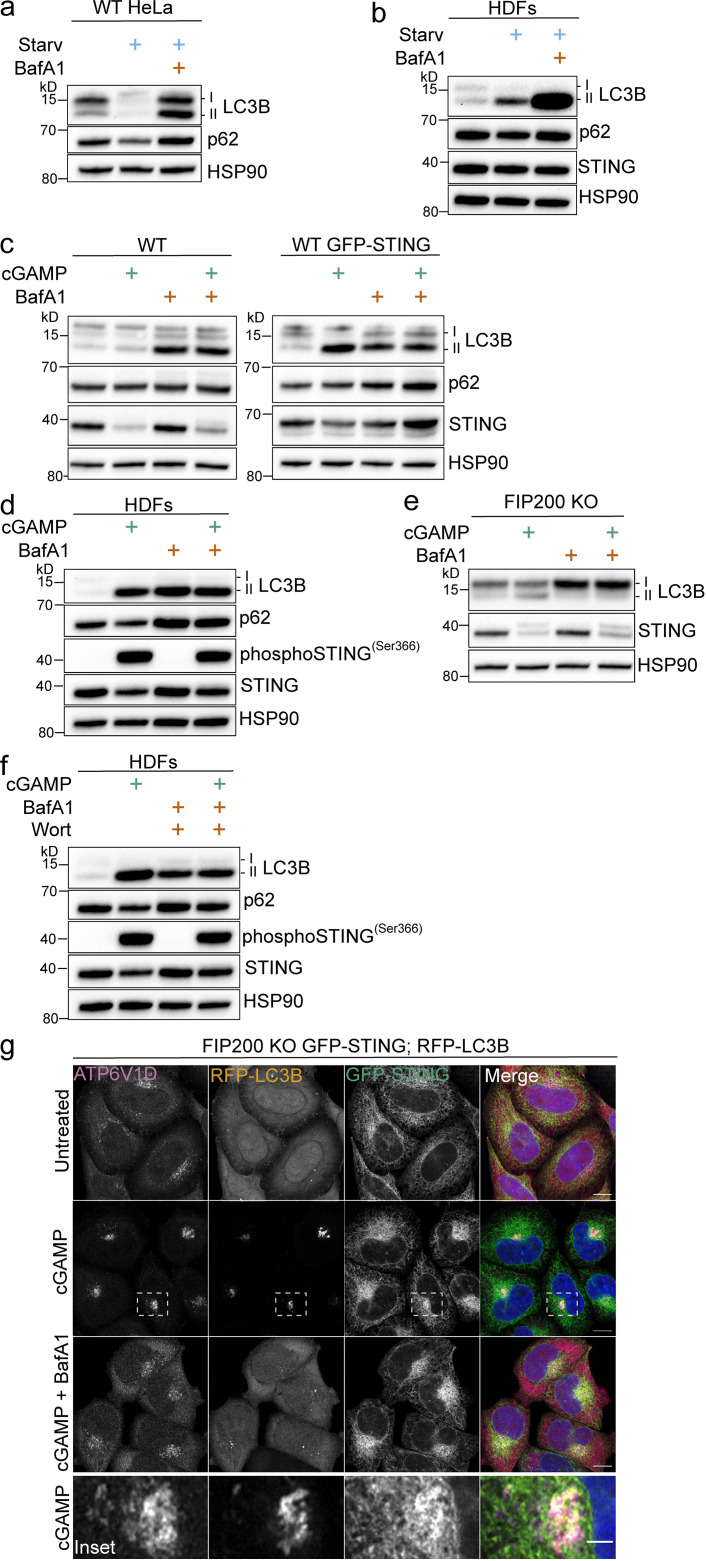
**cGAMP induces LC3B lipidation and redistribution of the V1 complex in the perinuclear region that is sensitive to pharmacological inhibition of the V-ATPase. (a and b)** WT HeLa cells and primary HDFs were incubated in starvation media (HBSS with Ca^2+^ and Mg^2+^) alone and with 100 nM BafA1 for 8 h (a) and 4 h (b). **(c)** WT HeLa cells and WT HeLa cells with stable expression of GFP-STING were incubated with 15 µg/ml (WT) or 60 µg/ml (WT GFP-STING) cGAMP alone and with 100 nM BafA1 for 8 h. All the following experiments were treated the same unless specified. **(d)** HDFs were incubated with 60 µg/ml cGAMP alone and with BafA1 for 4 h.** (e)** FIP200 KO HeLa cells were incubated with cGAMP alone and with BafA1. **(f) **HDFs were incubated with 60 µg/ml cGAMP alone, with BafA1 and 200 nM wortmannin (Wort) alone, and with cGAMP, BafA1, and Wort for 4 h. **(g)** Representative Airyscan-processed confocal imaging of FIP200 KO HeLa cells with stable expression of RFP-LC3B and GFP-STING treated with cGAMP (60 µg/ml) alone and with BafA1, fixed, and immunostained for endogenous ATP6V1D. Scale bar, 10 µm, 2 µm (inset). Each Western blotting experiment was independently replicated three times. Starv, starvation.

**Figure 3.**
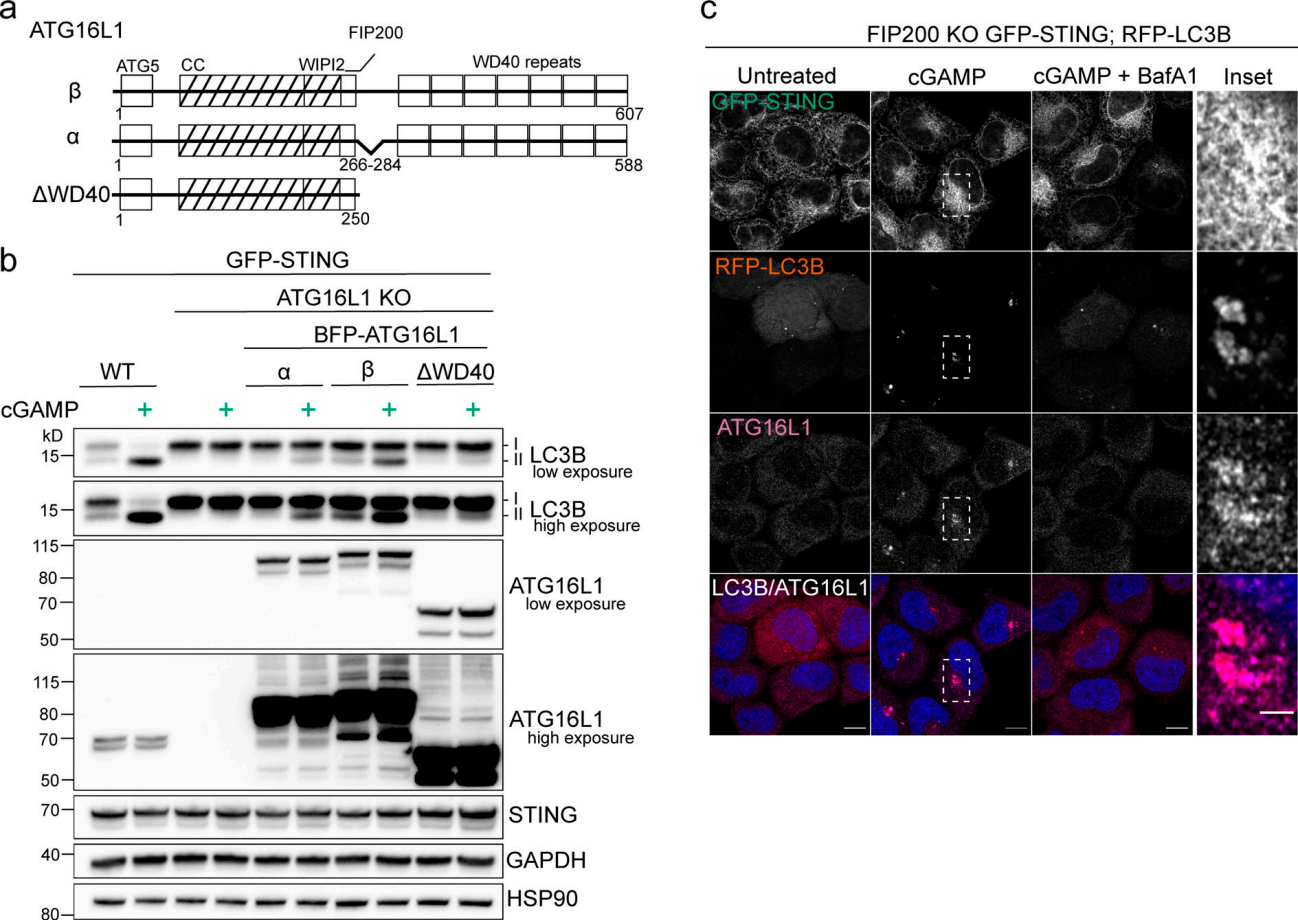
**STING activation induced LC3B lipidation is mediated by the WD40 domain of ATG16L1. (a)** Schematic representation of domains in the ATG16L1 isoforms (α and β) and ΔWD40 assessed in d. CC, coiled-coil. WIPI2 and FIP200 indicate interacting regions. **(b)** WT and ATG16L1 KO HeLa cells stably expressing GFP-STING and BFP-ATG16L1-α, BFP-ATG16L1-β, or ATG16L1-ΔWD40 were treated with 60 µg/ml cGAMP for 8 h. **(c)** Representative Airyscan-processed confocal imaging of FIP200 KO HeLa cells with stable expression of RFP-LC3B and GFP-STING treated with cGAMP (60 µg/ml) alone and with 100 nM BafA1 for 8 h, fixed, and immunostained for endogenous ATG16L1. Scale bar, 10 µm, 2 µm (inset). Each Western blotting experiment was independently replicated three times.

**Figure S2.**
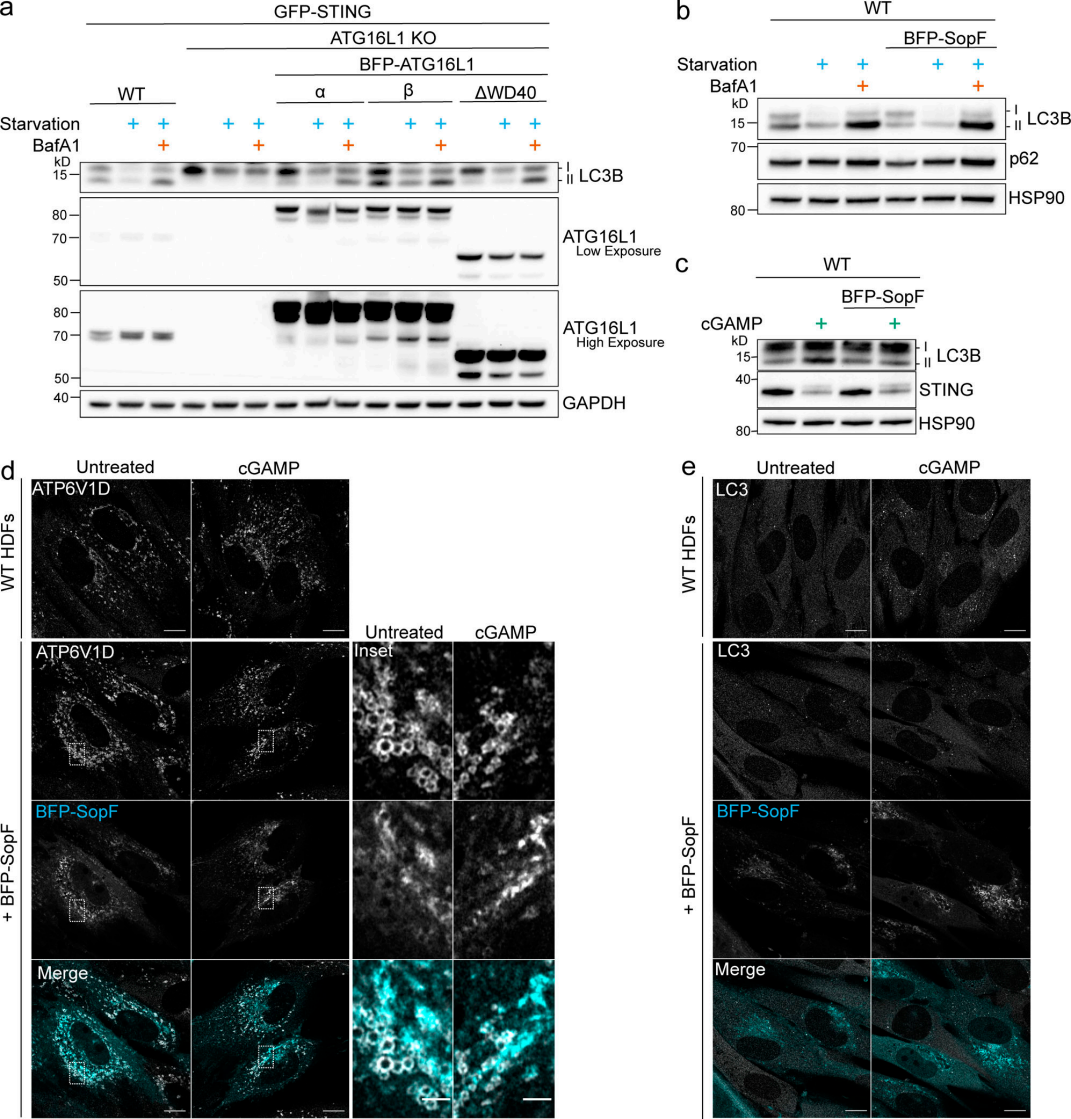
**Experiments supplementary to Figs. 3 and 5.** **(a)** WT and ATG16L KO HeLa cells with stable expression of GFP-STING and BFP-ATG16L-α, BFP-ATG16L-β, or ATG16L-ΔWD40 were incubated in starvation media (HBSS with Ca^2+^ and Mg^2+^) alone and with 100 nM BafA1 for 8 h. All the following experiments use the same conditions unless specified. **(b and c)** WT HeLa cells and WT HeLa cells with stable expression of BFP-SopF were incubated in starvation media alone and with BafA1 for 4 h (b) and treated with 15 µg/ml cGAMP for 8 h (c). Western blotting experiments were independently replicated three times. **(d and e)** Representative Airyscan confocal imaging of primary HDFs and HDFs with stable expression of BFP-SopF were treated with 60 μg/ml cGAMP for 4 h, fixed, and immunostained for endogenous ATP6V1D (d) or LC3 (e). Scale bar, 10 µm.

To examine the autophagic elimination of lipidated LC3B, we combined treatment of cGAMP with bafilomycin A1 (BafA1), a commonly used inhibitor of autophagy flux that both neutralizes lysosomal pH and blocks autophagosome-lysosome fusion (Yoshimori et al., 1991). BafA1 treatment promotes the accumulation of undegraded lipidated LC3B from starvation-induced autophagy in both WT HeLa and primary HDFs (Fig. 2, a and b), as well as in primary MEFs (Fig. S1 g). However, the accumulation of lipidated LC3B with BafA1 combined with cGAMP did not increase above that of basal LC3B lipidation with BafA1 alone in WT HeLa cells with either endogenous STING or stable overexpression of GFP-STING (Fig. 2 c), nor with endogenous STING in primary HDFs (Fig. 2 d) or primary MEFs (Fig. S1 h). To reduce the contribution of lipidated LC3B from basal autophagy, we treated FIP200 KO HeLa cells with cGAMP and found that the addition of BafA1 completely blocked STING-dependent LC3B lipidation (Fig. 2 e). Similarly, the addition of wortmannin to inhibit LC3B lipidation from basal autophagy in primary HDFs and MEFs revealed a block in cGAMP-induced LC3B lipidation by BafA1 (Fig. 2 f and Fig. S1 i, respectively). To further determine whether the block in cGAMP-induced LC3B lipidation was specific to BafA1 or was dependent on the neutralization of lysosomal pH, we assessed the effect of chloroquine, which increases the pH in lysosomal compartments as a weak base independently of the V-ATPase. Chloroquine treatment in WT HeLa cells caused accumulation of lipidated LC3B from basal autophagy, as expected, but did not prevent STING-dependent LC3B lipidation in FIP200 KO HeLa cells (Fig. S1 j), indicating that the block of cGAMP-induced LC3B lipidation by BafA1 is not due solely to neutralization of lysosomal pH. The V1 complex of the V-ATPase is both soluble in the cytosol and docked onto the integral membrane V0 complex (Cotter et al., 2015; Forgac, 2007). cGAMP treatment induced the redistribution of V1D, a subunit of the V1 complex, from a diffuse to a denser formation in the perinuclear region, colocalized with RFP-LC3B foci in the proximity of perinuclear GFP-STING in FIP200 KO HeLa cells (Fig. 2 g). The addition of BafA1 with cGAMP treatment blocked the redistribution of V1D as well as RFP-LC3B foci formation (Fig. 2 g), supporting the role of the V-ATPase in STING-dependent LC3B lipidation. RFP-LC3B does not colocalize with endogenous LAMP2 (Fig. S1 k), indicating that LC3B is not associated with lysosomes per se. Interestingly, the V-ATPase has been implicated in mediating ULK complex–independent forms of LC3B lipidation induced by lysosomotropic agents, such as chloroquine and monensin, as well as phagocytic events such as LC3-associated phagocytosis (LAP), entosis, and xenophagy (Fletcher et al., 2018; Xu et al., 2019). This noncanonical LC3B lipidation is mediated by direct recruitment of ATG16L1 to the V-ATPase via its WD40 domain, bypassing the need for upstream ULK complex initiation (Fletcher et al., 2018; Xu et al., 2019). The WD40 domain of ATG16L1, which is not conserved in yeast, has been consistently shown to be dispensable for starvation-induced autophagy (Fletcher et al., 2018; Fujita et al., 2009; 2013; Mizushima et al., 2003; Xu et al., 2019). We examined the ability of full-length ATG16L1 (ATG16L1-β, 607 aa isoform; Fig. 3 a) and a mutant lacking the WD40 domain (ΔWD40, aa 1–250; Fig. 3 a) to rescue LC3B lipidation in ATG16L1 KO HeLa cells. As previously shown, Atg16L1-β and Atg16L1-ΔWD40 both rescue starvation-induced LC3B lipidation in ATG16L1 KO cells (Fig. S2 a; Fletcher et al., 2018; Xu et al., 2019). However, Atg16L1-ΔWD40 failed to rescue LC3B lipidation induced by cGAMP, in contrast to Atg16L1-β (Fig. 3 b), indicating that the WD40 domain is required for STING-dependent LC3B lipidation. We also examined the requirement for an 18–amino acid region between the FIP200-binding and WD40 domains that has been reported to be important for ULK complex–independent LC3B lipidation at endolysosomes induced by the sodium ionophore, monensin (Lystad et al., 2019). The α isoform was less effective in rescuing starvation-induced LC3B lipidation (Fig. S2 a) as well as in rescuing cGAMP-induced LC3B lipidation compared with the β isoform in ATG16L1 KO cells (Fig. 3 b). Further, we assessed the localization of endogenous ATG16L1 in FIP200 KO cells. Treatment of cells with cGAMP induced the translocation of ATG16L1 to the perinuclear region, which colocalized with GFP-LC3B foci in the proximity of perinuclear mCh-STING (Fig. 3 c). BafA1 treatment blocked this perinuclear ATG16L1 translocation (Fig. 3 c), consistent with the BafA1 prevention of V1D redistribution and LC3B foci formation (Fig. 2 g), as well as LC3B lipidation detected by Western blotting (Fig. 2 e). The requirement of the ATG16L1 WD40 domain and its ability to conjugate LC3B independently of the ULK complex further distinguishes STING-dependent LC3B lipidation from canonical autophagy (Fujita et al., 2013; Mizushima et al., 2003).

**Figure 4.**
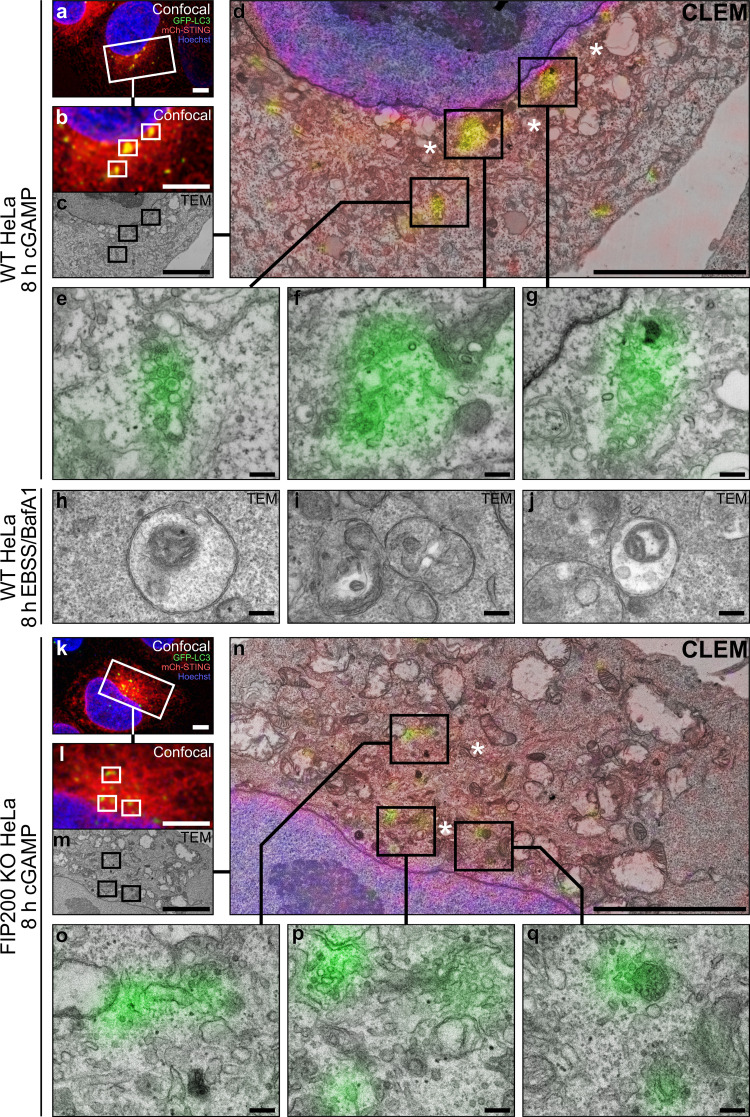
**GFP-LC3B is targeted to perinuclear single-membrane vesicles upon STING activation by cGAMP.** **(a)** Confocal image data (deconvolved; maximum projected) acquired from WT HeLa cells expressing GFP-LC3B (green) and mCh-STING (red), and stained with Hoechst 33342 (blue) after 8 h cGAMP (60 µg/ml) incubation, before correlative imaging of the indicated region of interest (white inset frame). **(b–d)** Spatial alignment between the (b) optical and (c) electron micrographs acquired within the region indicated in a, (d) with direct overlay between imaging modalities. **(e–g)** Correlative micrographs of GFP-LC3B–positive structures detected from d. **(h–j)** Representative electron micrographs of starvation induced autophagosomal structures in WT HeLa cells after 8 h incubation with Earle's Balanced Salt Solution in the presence of BafA1 (100 nM). **(k)** Confocal image data (deconvolved; maximum projected) acquired from FIP200 KO HeLa cells expressing GFP-LC3B and mCh-STING, stained with Hoechst 33342 after 8 h cGAMP (60 µg/ml) incubation. **(l–n)** Spatial alignment between (l) optical and (m) electron micrographs acquired at region indicated in k, (n) displayed with direct overlay between micrographs. **(o–q)** Selected examples of GFP-LC3B–positive structures detected from n. Portions of the Golgi apparatus indicated by asterisk. Scale bars, a–d and k–n, 5 µm; e–j and o–q, 200 nm. EBSS, Earle's Balanced Salt Solution.

Collectively, our data suggest that LC3B lipidation induced by cGAMP activation of STING is related to noncanonical forms of LC3B conjugation onto the outer leaflet of single-membrane vesicles, rather than double-membrane autophagosomes (Fletcher et al., 2018; Florey et al., 2011, 2015; Gammoh et al., 2013; Jacquin et al., 2017; Martinez et al., 2011, 2015; Sanjuan et al., 2007). Although double-membrane structures observed by EM have been previously reported after cGAMP treatment in both WT and ULK1/2 double KO MEFs, it is unclear whether these structures are associated with STING activation–induced LC3B foci (Gui et al., 2019). We used correlative light EM (CLEM) to identify GFP-LC3B–labeled structures in WT and FIP200 KO HeLa cells following cGAMP treatment and to clarify whether LC3B is localized to single- or double-membrane structures. CLEM identification of GFP-LC3B–labeled structures in the perinuclear region after cGAMP treatment revealed that LC3B is localized with single-membrane vesicles in both WT (Fig. 4, a–g) and FIP200 KO HeLa (Fig. 4 k–q). These single-membrane structures are distinct from canonical autophagosomes induced by starvation detected by transmission EM (TEM; Fig. 4, h–j). No double-membrane vesicles or autophagosome-like structures were observed underlying GFP-LC3B foci, or elsewhere in the cytoplasm, in single slices of cGAMP treated WT and FIP200 KO cells. The absence of double membrane structures in FIP200 KO HeLa cells is consistent with the requirement of FIP200 for initiation of autophagosome formation and previous EM reports in FIP200 KO MEFs (Hara et al., 2008; Kishi-Itakura et al., 2014). The identified single-membrane vesicles labeled with GFP-LC3B may be consistent with previous EM analysis observing STING localization to perinuclear single-membrane vesicles following transfection with double-stranded DNA (dsDNA; Gonugunta et al., 2017; Saitoh et al., 2009). The GFP-LC3B–labeled single-membrane vesicles are also found in proximity of Golgi structures (asterisks in Fig. 4, d and n), which is consistent with the perinuclear localization of LC3B and STING (Fig. 1 f, Fig. 2 g, and Fig. 3 c) and aligns with previous reports of STING colocalization with Golgi markers upon trafficking (Dobbs et al., 2015; Gonugunta et al., 2017; Gui et al., 2019; Ishikawa et al., 2009). The CLEM identification of GFP-LC3B–positive single-membrane structures (Fig. 4) indicates that STING-dependent LC3B lipidation occurs on perinuclear single-membrane vesicles, more so than double-membrane autophagosomes.

**Figure 5.**
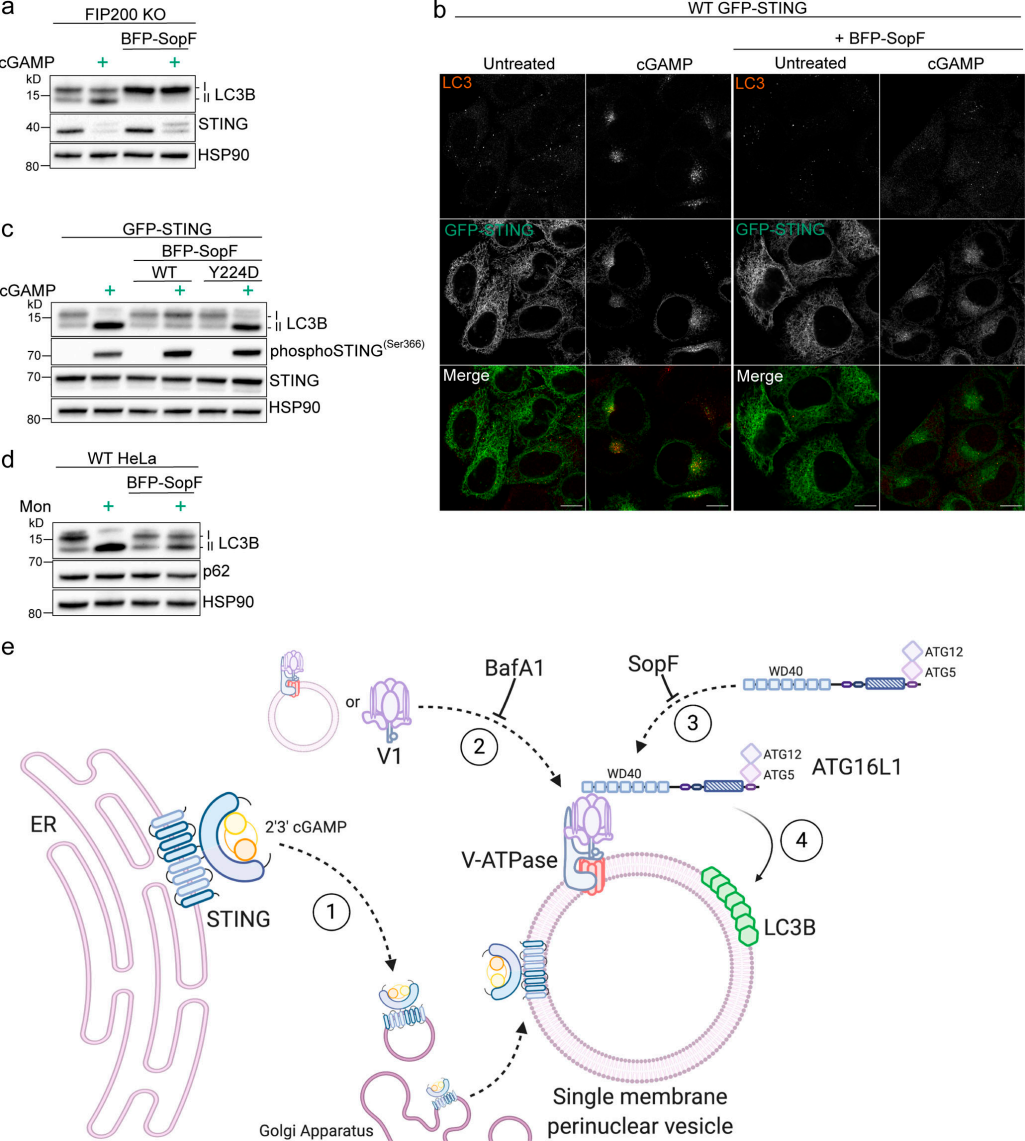
**The V-ATPase targeting bacterial effector, SopF, blocks STING-mediated LC3B lipidation and perinuclear foci formation. (a)** FIP200 KO HeLa cells and FIP200 KO HeLa cells with stable expression of mammalian codon optimized BFP-SopF were incubated with 15 µg/ml cGAMP for 8 h. **(b)** Representative Airyscan confocal imaging of WT HeLa cells with stable expression of GFP-STING alone and with GFP-STING and BFP-SopF were treated with 60 µg/ml cGAMP for 8 h, fixed, and immunostained for endogenous LC3. Scale bar, 10 µm. **(c)** WT HeLa cells with stable expression of GFP-STING alone and with either stable expression of BFP-SopF or BFP-SopF-Y224D were incubated with 60 µg/ml cGAMP for 8 h. **(d)** WT HeLa cells and WT HeLa cells with stable expression of BFP-SopF were incubated with 100 μM monensin for 1 h. Western blotting experiments were independently replicated three times. **(e)** Model of VAIL onto single-membrane perinuclear vesicles induced by cGAMP activation of STING created with BioRender.com. (1) cGAMP-activated STING translocates from the ER to the Golgi apparatus to colocalize at or around single-membrane perinuclear vesicles. (2) The V1 complex docks to resident V0 domains in perinuclear vesicles, or vesicles with assembled V-ATPases redistribute to a denser formation in the perinuclear region. This process is blocked by BafA1. (3) ATG16L1 is recruited to interact with the V-ATPase via its WD40 domain (as reported by Xu et al., 2019). (4) LC3B is conjugated to phosphatidylethanolamine on single-membrane perinuclear vesicles by the ATG16L1-ATG5-12 complex. This process is inhibited by BafA1 and SopF. Mon, monensin.

Recently, a bacterial effector required for *Salmonella typhimurium* virulence, SopF, was shown to inhibit LC3B lipidation of the *Salmonella*-containing vacuole by ADP-ribosylation of the C subunit in the V0 complex of the V-ATPase (Xu et al., 2019). In contrast to canonical xenophagy, but similar to STING-induced LC3B lipidation, the SopF-sensitive LC3B lipidation induced by *Salmonella* infection was inhibited by BafA1 and mediated by the C-terminal WD40 domain of Atg16L1 independently of FIP200. Importantly, ADP ribosylation of the V-ATPase by SopF did not affect endolysosomal pH or autophagosome–lysosome fusion, presenting SopF as a potential tool to impair V-ATPase–mediated LC3B lipidation without disrupting lysosomal acidification. As previously reported (Xu et al., 2019), expression of codon-optimized BFP-SopF in HeLa cells did not cause basal lipidation of LC3B or block the degradation of lipidated LC3B induced by starvation (Fig. S2 b). However, SopF expression robustly inhibited cGAMP-induced LC3B lipidation in both WT (Fig. S2 c) and FIP200 KO HeLa cells (Fig. 5 a). Importantly, endogenous STING degradation upon cGAMP treatment was not blocked with the expression of SopF in WT (Fig. S2 c) or FIP200 KO HeLa cells (Fig. 5 a), indicating that STING activation and degradation were not perturbed. SopF expression also did not affect trafficking of GFP-STING to the perinuclear region but blocked the formation of perinuclear LC3 foci induced by cGAMP in WT cells (Fig. 5 b). Stable expression of SopF in primary HDFs demonstrated partial colocalization with endogenous V1D (Fig. S2 d), as previously reported (Xu et al., 2019), and reduced LC3 punctae formation induced by cGAMP (Fig. S2 e) consistently with HeLa cells (Fig. 5 b). It is important to note that endogenous V1D labeling in these primary human cells displays a more vesicular and broad cytosolic distribution compared with HeLa cells. Thus, it is difficult to determine whether there is a distinct change in the distribution of V1D as observed in FIP200 KO HeLa cells (Fig. 2 g). Xu et al. (2019) identified a mutation, Y224D, in SopF that abolishes the ability of SopF to block LC3B association with *Salmonella* upon infection. Consistently, stable expression of SopF-Y224D did not block cGAMP-induced LC3B lipidation in WT HeLa cells stably expressing GFP-STING (Fig. 5 c). Further, consistent with the V-ATPase–dependent mechanism of LC3B lipidation induced by monensin, we found that SopF expression also blocks LC3B lipidation upon monensin treatment (Fig. 5 d; Florey et al., 2015). These results corroborate our findings that BafA1 blocks STING-dependent LC3B lipidation, further supporting the role of the V-ATPase in LC3B lipidation induced by cGAMP activation of STING.

**Figure S3.**
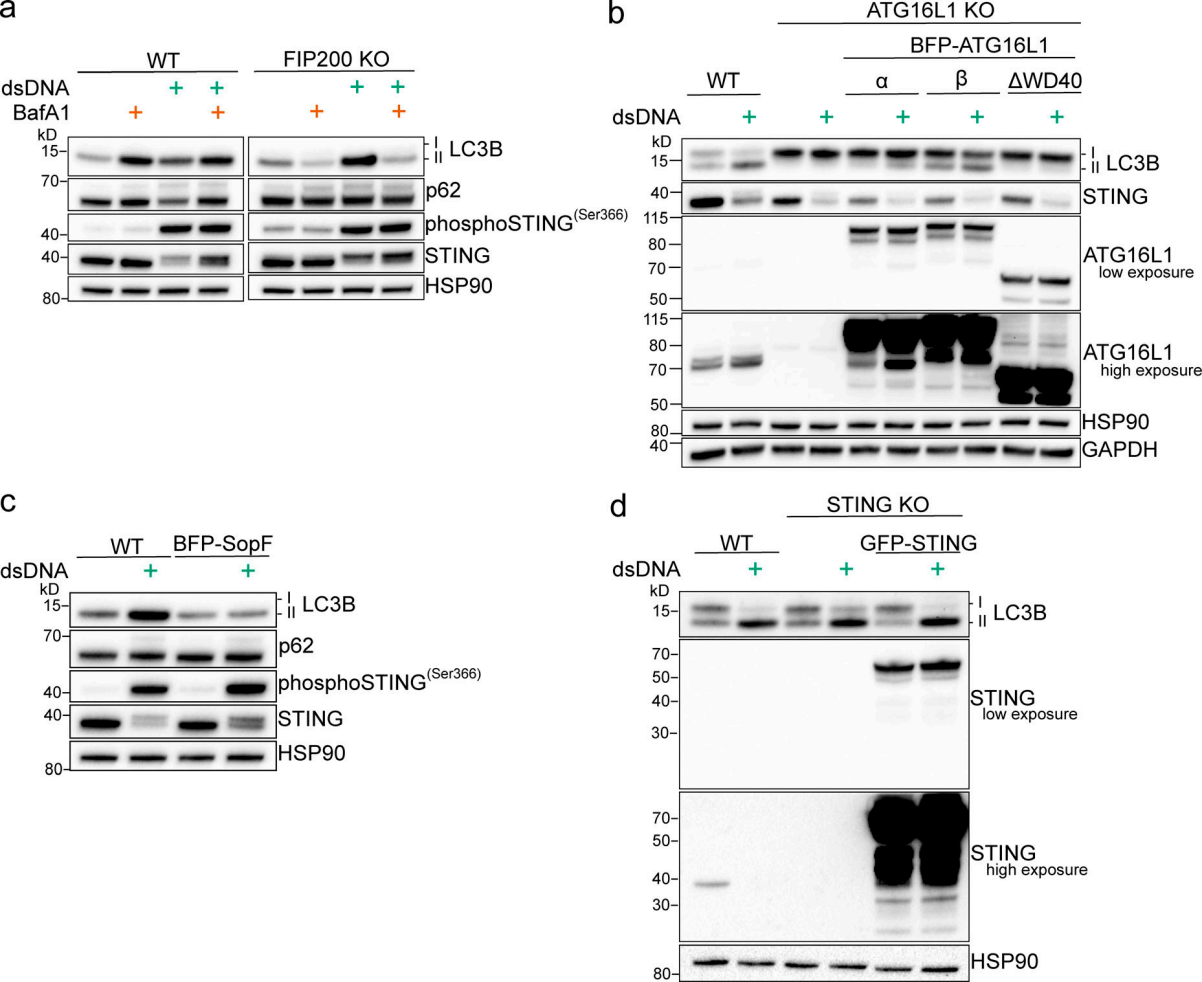
**VAIL is induced by transfection of poly(dA:dT) independently of STING.** **(a)** WT and FIP200 KO HeLa cells were transfected with 4 μg/ml poly(dA:dT) (dsDNA) and Lipofectamine 2000 at (1:1.5 μg DNA: μl Lipofectamine) alone and with BafA1 (100 nM) for 4 h. All the following experiments were treated the same unless specified. **(b)** WT and ATG16L KO HeLa cells with stable expression of GFP-STING and BFP-ATG16L-α, BFP-ATG16L-β, or ATG16L-ΔWD40 were transfected with dsDNA. **(c) **WT HeLa cells and WT HeLa cells with stable expression of BFP-SopF were transfected with dsDNA. **(d)** WT, STING KO, and STING KO HeLa cells stably expressing GFP-STING were transfected with dsDNA.

The data presented in this study show that cGAMP induces STING-dependent lipidation of LC3B onto single-membrane perinuclear vesicles that is mediated by ATG16L1 via the WD40 domain rather than machinery required for initiation of autophagosome formation in canonical autophagy. Further, we show that cGAMP induces redistribution of the V1 complex of the V-ATPase in the perinuclear region and that ATG16L1 recruitment and LC3B lipidation are mediated by the V-ATPase. These data provide key mechanistic insights into LC3B lipidation induced by STING activation, revealing a mechanism, distinct from autophagy, that we call V-ATPase–ATG16L1—induced LC3B lipidation (VAIL; Fig. 5 e). V-ATPase–sensitive LC3B lipidation that occurs at single membranes rather than autophagosomes has been previously observed on Golgi-related and endolysosomal compartments, as well as on phagocytic vesicles containing bacteria, fungi particles, or apoptotic cells in a process known as LAP (Fletcher et al., 2018; Florey et al., 2011, 2015; Gammoh et al., 2013; Jacquin et al., 2017; Martinez et al., 2011, 2015; Sanjuan et al., 2007; Xu et al., 2019). Interestingly, LAP is associated with the activation of Toll-like receptors (TLRs), a collection of PRRs that also initiate the type I IFN response upon recognition of various pathogen-related molecular features, including nucleic acids (Hayashi et al., 2018; Henault et al., 2012; Sanjuan et al., 2007). TLR activation by phagocytosis of latex beads coated with lipopolysaccharide, zymosan, or DNA-immune complexes induces LC3B lipidation on the phagocytic vesicle that is also mediated by the ATG16L1-WD40 domain and is sensitive to V-ATPase inhibition (Fletcher et al., 2018; Florey et al., 2015). It is important to note that the mechanisms of STING-dependent LC3B lipidation are distinct from LAP, as LAP requires PI3K activity and formation of a phagocytic vesicle, whereas STING-dependent LC3B lipidation does not (Fig. 1, c and d; and Fig. S1 d; Henault et al., 2012; Martinez et al., 2011, 2015; Sanjuan et al., 2007). Rather, this study shows that V-ATPase–mediated LC3B lipidation can be induced directly through the binding and activation of STING by its ligand, 2′3′-cGAMP. We also assessed transfection of poly(dA:dT), a synthetic dsDNA ligand that can induce synthesis of endogenous 2′3′-cGAMP by cGAS to activate STING (Wu et al., 2013). Poly(dA:dT) transfection induced V-ATPase–ATG16L1–WD40 domain—mediated LC3B lipidation that is independent of FIP200 and sensitive to SopF, similarly to cGAMP (Fig. S3, a–c). However, this LC3B lipidation was not fully dependent on STING in HeLa cells (Fig. S3 d). Poly(dA:dT) is known to activate multiple PRR pathways, and transfection reagents may cause damage to endolysosomes; therefore, we caution the future use of this ligand and DNA:transfection complexes to study STING-dependent VAIL (Kobayashi et al., 2010; Unterholzner, 2013). Nonetheless, the link between both TLR and STING activation and the induction of V-ATPase–sensitive LC3B lipidation on single-membrane vesicles suggests it may represent a unified cellular response of innate immune pathways.

We do not know how cGAMP activation of STING induces V1 complex redistribution and ATG16L1-WD40 translocation to mediate LC3B lipidation at perinuclear single-membrane vesicles. Xu et al. (2019) reported that the WD40 domain of ATG16L1 interacts with both V1 and V0 complexes of the V-ATPase during SopF-sensitive LC3B lipidation on the *Salmonella*-containing vacuole. Similarly, Atg16L1 translocation to the perinuclear region does correlate with the spatial redistribution of the V1 complex, where they colocalize with LC3B foci following cGAMP treatment (Fig. 2 g and Fig. 3 c). V-ATPase complexes are localized throughout the secretory pathway and regulate pH in various vesicular and organellar compartments, including the Golgi apparatus, endosomes, and lysosomes (Cotter et al., 2015; Forgac, 2007). Recent studies have reported that endolysosomal damage induces ATG16L1-WD40–mediated LC3B lipidation via the V-ATPase due to ionic imbalances and swelling caused by the sodium ionophore activity of monensin (Florey et al., 2015). Although STING has been shown to traffic to endolysosomes, where it is degraded in a RAB7– and V-ATPase–dependent manner, LC3B lipidation induced by STING activation has been reported to occur at ER-Golgi intermediate compartments (Dobbs et al., 2015; Gonugunta et al., 2017; Gui et al., 2019; Konno et al., 2013). The single-membrane perinuclear vesicles associated with GFP-LC3B following cGAMP treatment identified using CLEM (Fig. 4) may support ER-Golgi intermediate compartments as the target membrane; however, it is difficult to rule out whether these vesicles are derived from trafficking between the ER and Golgi or are endolysosomal in origin.

Gui et al. (2019) found that exogenous expression of RavZ, an effector expressed by *Legionella pneumophila* that is known to de-lipidate LC3B and promote its survival in host cells, reduces the effectiveness of cGAMP to limit herpes simplex virus–1 replication (Choy et al., 2012). Thus, the lipidation of LC3B onto single-membrane vesicles induced by STING activation (Fig. 4) may facilitate cGAMP-mediated viral defense in the host cell. Furthermore, SopF promotes survival of *S. typhimurium* by preventing LC3B lipidation mediated specifically by the ATG16L1-WD40 interaction with the V-ATPase (Xu et al., 2019). VAIL may represent a general mechanism for cell-autonomous innate immunity that includes anti-viral (Gui et al., 2019) and anti-bacterial host defense (Xu et al., 2019). How V-ATPase–ATG16L1—induced LC3B lipidation may accomplish host defense requires further investigation. LAP is thought to promote fusion with lysosomes and/or endosomes to facilitate elimination of phagocytosed material (Henault et al., 2012; Martinez et al., 2015). Consistently, ATG8s have been shown to mediate membrane fusion in vitro and in vivo (Nakatogawa et al., 2007; Nguyen et al., 2016). V-ATPase–ATG16L1—induced LC3B lipidation may similarly promote lysosomal fusion for elimination of viruses and bacteria or to impede pathogen trafficking through the endocytic or secretory pathways.

Importantly, as LC3B lipidation is a conserved feature of the STING homologue in *N. vectensis*, our results suggest that LC3B lipidation at single membranes via the V-ATPase and ATG16L1 WD40 domain may be an important function of STING predating the IFN response (Gui et al., 2019). Consistently, an ATG16L1 homologue has been found in *N. vectensis* that contains conserved C-terminal WD repeats (Bajagic et al., 2017). The emergence of the ATG16L1 WD40 domain in metazoa may also support an evolutionarily conserved function of ATG16L1 to mediate the recruitment of the LC3B conjugation machinery independently of autophagy. Thus, VAIL stimulated by cyclic dinucleotide activation of STING may represent an evolutionarily ancient mechanism of cell-autonomous innate immunity.

## Materials and methods

### Cell culture, reagents, and antibodies

HeLa cells (CCL-2) and primary neonatal HDFs (PCS-201-010) were purchased from American Type Culture Collection. Primary MEFs were obtained as previously described (Narendra et al., 2008). Cells were cultured in DMEM (Thermo Fisher Scientific; 31053028) supplemented with 10% (vol/vol) FBS (Sigma-Aldrich), 1 mM sodium pyruvate (Thermo Fisher Scientific; 11360070), and 2 mM GlutaMAX (Thermo Fisher Scientific; 35050061). For starvation, cells were incubated in HBSS supplemented with Mg^2+^ and Ca^2+^ (Caisson Labs). All cell lines were routinely tested for mycoplasma using the PlasmoTest kit (InvivoGen).

2′3′-cGAMP was purchased from Chemitek (used at 15–60 ug/ml), and poly(dA:dT) was purchased from Invivogen (used at 4 μg/ml). Lipofectamine 2000 was purchased from Thermo Fisher Scientific (used at 1:1.5 μg DNA: μl lipofectamine. Information for pharmacological inhibitors is as follows: BafA1 (Sigma-Aldrich; used at 100 nM), chloroquine (Sigma-Aldrich; used at 20 μM), wortmannin (Calbiochem; used at 200 nM), and VPS34-IN1 (Selleck Chemicals; used at 300 nM).

For Western blotting, the following antibodies were used: rabbit anti-LC3b (Sigma-Aldrich; L7543), mouse anti-SQSTM1/p62 (Abnova; H00008878-M01), rabbit anti-STING (D2P2F; CST; 13647), rabbit anti-HSP90(α/β) (H-114; SCBT; sc-7947), mouse anti-WIPI2 (Abcam; ab105459), rabbit anti-ATG16L1 (D6D5; CST; 8089), and rabbit anti-GAPDH (Sigma-Aldrich; G9545). For immunofluorescence, the following antibodies were used: rabbit anti-ATP6V1D (Abcam; ab157458), rabbit anti-LC3 (MBL International; PM036), rabbit anti-ATG16L1 (D6D5; CST; 8089), and Alexa Fluor 568, 594, and 647 conjugated secondary antibodies (Life Technologies).

### KO cell lines

STING KO HeLa cells and corresponding WT HeLa cells were a kind gift from F. Van Kuppeveld (Utrecht University, Utrecht, The Netherlands) and were generated as described in Langereis et al. (2015). FIP200 KO HeLa cells were previously described in Vargas et al. (2019), and N/O/Tx TKO HeLa cells were previously described in Lazarou et al. (2015). For generation of ATG16L1 KO and WIPI2 and WIPI4 KO HeLa cells with CRISPR/Cas9, gRNAs were cloned into pSpCas9(BB)-2A-Puro (PX459) V2.0 (Addgene plasmid no. 62988). HeLa cells were transfected with the gRNA plasmids and treated with 1 μg/ml puromycin 2 d to enrich transfected cells, which were then diluted and placed into 96-well plates for single colonies. PCR screening was performed, and positive clones were further Sanger-sequenced and analyzed with the ICE (Inference of CRISPR Edits) online analysis tool (https://ice.synthego.com/#/) to identify the frame-shifting deletions or insertions (InDels) and finally by Western blot. Details of each gRNA sequence, PCR genotyping, and InDels in each KO clone are shown in Table S1.

### Cloning and stable cell line generation

Modified pHAGE (plasmid HIV-1 Alex Gustavo George Enhanced) vectors were used for cloning either by Gateway recombination cloning technology (Gate; Thermo Fisher Scientific) or Gibson assembly cloning kit (New England Biolabs). The ORF of human STING cDNA (NM_198282.4) was PCR-amplified from Addgene plasmid #102598 and cloned into a linearized pDONR-223 donor vector. The pDONR-223-STING vector was then cloned into a pHAGE-Gate(way) destination vector as described in the manufacturer’s protocol. All other pHAGE vector plasmids were generated by PCR amplification of ORF cDNA and ligation into the linearized pHAGE vectors using Gibson cloning (New England Biolabs) according to the manufacturer’s protocol. All constructs used or generated in this study were validated by Sanger sequencing, and complete plasmid sequences are available upon request.

Stable cell lines were generated by lentivirus infection. Virus packaging and host cell infection were previously described in detail (Wang, 2020). Cells were sorted based on expression of tagged fluorescent proteins using FACS to obtain homogenous cell populations at optimal protein expression.

### Western blotting

Cells plated in 12-well plates were washed with ice-cold HBSS, lysed directly in ice-cold 1× Lithium Dodecyl Sulfate sample buffer (Thermo Fisher Scientific) supplemented with cOmplete Protease Inhibitor cocktail (Roche), and boiled immediately at 99°C for 10 min. Protein quantitation per sample was obtained using the Pierce BCA protein assay kit (Thermo Fisher Scientific). DTT (Sigma-Aldrich) was then added to each sample at a final concentration of 100 mM before gel loading. 10–20 μg of cell lysate per sample was loaded into 4–12% Bis-Tris gels (GenScript) and separated in MES or MOPs buffer (GenScript). Separated proteins were then transferred onto 0.45 µm nitrocellulose (BioRad) or 0.45 µm polyvinylidene difluoride (Thermo Fisher Scientific) membranes in Towbin’s buffer. Membranes were blocked in 5% milk in 1× PBS supplemented with Tween-20 at RT for 1 h before primary antibody incubation in 3% BSA (Tocris) in PBS supplemented with Tween-20 at 4°C overnight. Membranes were washed and then incubated in appropriate HRP secondaries (Cell Signaling) for 1 h at RT before signal development in Amersham ECL (GE Healthcare) or SuperSignal West Femto ECL (Thermo Fisher Scientific). ECL signal was detected using a ChemiDoc Imaging System (BioRad) and analyzed using the ImageLab (BioRad) software.

### Immunofluorescence and confocal microscopy

Cells plated onto no. 1.5 pretreated glass coverslips (NeuVitro) in 12-well plates or no. 1.5 chambered coverglass (Nunc LabTek II) were fixed for 10–15 min at 37°C in prewarmed 4% PFA (Electron Microscopy Sciences) or ice-cold methanol (Fisher Chemical) at −20°C for 10 min. After fixation, cells that were fixed with PFA were washed with 1× PBS and permeabilized with 0.5% Triton X-100 in 1× PBS for 5 min before blocking in 3% goat serum and 1% BSA with or without 0.1% Tween 20 in 1× PBS for 1 h. Cells fixed with methanol were washed with 1× PBS and then blocked in 3% goat serum, 1% BSA, and 0.3% Triton X-100 for 1 h. Both PFA- and methanol-fixed cells were then incubated with primary antibodies diluted in blocking buffer overnight at 4°C, washed with 1× PBS, and incubated with secondary antibodies diluted in blocking buffer for 1 h at RT. After final washing, coverslips were mounted on to microscope slides (Thermo Fisher Scientific) using Fluoromount-G with DAPI (Southern Biotech) or Vectashield (Vector Labs) mounting media and stored at 4°C until imaging. Cells plated in a chambered coverglass were imaged immediately following staining.

Fixed and immunostained cells were imaged on a Zeiss LSM 880 Airyscan Confocal Microscope using a Zeiss 63× 1.4 NA Plan-Apochromat objective at RT. Z-stack images were acquired using Airyscan detectors and a piezo high-precision stage using the Zen software platform (Carl Zeiss Microscopy). Images were then processed for Airyscan 3D deconvolution using Zen software and prepared for publication using FIJI open source software (Schindelin et al., 2012). The "HiLo" range indicator look up table in FIJI was used to determine the minimal pixel saturation for each image, and linear adjustments to the upper limits of the display range were performed.

### CLEM and TEM

WT or FIP200 KO HeLa cells stably expressing GFP-LC3B and mCh-STING were grown for 48 h in a 35-mm 500-grid plastic-bottom μ-Dish (Ibidi) before 8 h incubation with cGAMP and primary fixation. The sample was fixed using prewarmed phosphate-buffered 4% PFA (1 h at 37°C) and stained with 2.5 µg/ml CellMask deep red plasma membrane stain (Thermo Fisher Scientific) and 1 µM Hoechst 33342 stain (Thermo Fisher Scientific) as per the manufacturer's instructions. The fixed sample was imaged using an inverted Leica SP8 confocal laser scanning microscope equipped with a 40×/1.10 objective (water immersion, HC PLAPO, CS2; Leica Microsystems, Inc.) equipped with a HyD hybrid detector (Leica Biosystems). The optical data (47-nm lateral voxel resolution; 160-nm axial pixel resolution) was deconvolved (fast classic maximum likelihood estimate; signal to noise of 20 for 20 iterations) using Huygens Professional (v15.10; Scientific Volume Imaging) for later alignment.

The imaged sample was post-fixed overnight with 2.5% glutaraldehyde in 0.1 M sodium cacodylate buffer at 4°C, rinsed twice with 0.1 M sodium cacodylate, and then incubated with ferricyanide-reduced osmium tetroxide (1% [wt/vol] OsO_4_, 1.5% [wt/vol] K3[Fe{CN}6], 0.1 M cacodylate buffer) for 2 h at 4°C. Microwave-assisted sample processing was employed in all subsequent stages using a BioWave Pro microwave system (Pelco). The sample was en bloc stained with 2% (wt/vol) aqueous uranyl acetate using three cycles of 100 W on for 120 s, off for 120s under vacuum, dehydrated by a graduated series of ethanol (50%, 70%, 90%, 100%, and 100%), then propylene oxide (100% and 100%) at atmospheric pressure (150 W for 40 s per stage), and infiltrated with a graduated series of Embed 812/Araldite 502 resin (25%, 50%, 75%, 100%, and 100%) in propylene oxide (250 W for 180 s per stage under vacuum). The resin was polymerized overnight at 60°C before relocation and extraction of the correlative target region, which was attached to a Embed 812/Araldite 502 resin block and polymerized overnight at 60°C for ultramicrotomy. The resin block was manually trimmed, then serial-sectioned to the target depth (∼1 µm from coverslip surface for both samples) using a 45° diamond knife (Diatome) on an Ultracut UCT ultramicrotome (Leica Biosystems). Sections (75 nm thick) cut from the target region were collected on 300-mesh Hex thin-bar copper grids, and stained at RT using 2% (wt/vol) aqueous uranyl acetate (10 min) and Reynolds lead citrate (3 min), before imaging on an JEM-1400PLUS transmission electron microscope equipped with a scientific Complementary Metal-Oxide Semiconductor (sCMOS) camera (JEOL). A montage of electron micrographs of the target cells was acquired, distortion-corrected (Kaynig et al., 2010), and then spatially aligned to the corresponding optical sections (depth of 1,280 nm for the WT sample; depth of 960 nm for the FIP200 KO sample), which were identified from the deconvolved confocal data by aligning filopodia to the CellMask deep-red stain. Spatial alignment between datasets was conducted using GIMP (GNU Image Manipulation Program, Version 2.8.16) as previously reported (Padman et al., 2014). All subsequent TEM images were aligned directly to the distortion-corrected TEM montage for display.

For TEM imaging of starvation-induced autophagosomes (Fig. 4, h–j), WT HeLa cells were cultured in a six-well plate for 48 h before incubation with Earle's Balanced Salt Solution starvation medium in the presence of 100 nM BafA1 (Bioaustralis) and fixed in prewarmed phosphate-buffered 4% PFA (1 h at 37°C). The cell monolayers were post-fixed and osmicated as described above, then scraped and pelleted in agarose for microwave-assisted sample processing. All subsequent processing stages were conducted as described above.

### Online supplemental material

Fig. S1 shows results from additional experiments supplementing Figs. 1 and 2. Fig. S2 shows results from additional experiments supplementing Figs. 3 and 5. Fig. S3 shows results from additional experiments using poly(dA:dT) as a STING stimulus. Table S1 lists information for all reagents and resources, as well as sequence information for all plasmids and CRISPR KO cell lines generated in the study.

## Supplementary Material

Table S1 lists information for all reagents and resources, as well as sequence information for all plasmids and CRISPR KO cell lines generated in the study.
